# Impact of synthetic and biological immunomodulatory therapy on the duration of 17DD yellow fever vaccine-induced immunity in rheumatoid arthritis

**DOI:** 10.1186/s13075-019-1854-6

**Published:** 2019-03-14

**Authors:** Clarissa de Castro Ferreira, Ana Carolina Campi-Azevedo, Vanessa Peruhype-Magalhāes, Jordana Grazziela Coelho-dos-Reis, Lis Ribeiro do Valle Antonelli, Karen Torres, Larissa Chaves Freire, Ismael Artur da Costa-Rocha, Ana Cristina Vanderley Oliveira, Maria de Lourdes de Sousa Maia, Sheila Maria Barbosa de Lima, Carla Magda Domingues, Andréa Teixeira-Carvalho, Olindo Assis Martins-Filho, Lícia Maria Henrique da Mota, on behalf of the Collaborative Group for Studies of Yellow Fever Vaccine

**Affiliations:** 1 Departamento de Reumatologia, Hospital Universitário de Brasília, Universidade de Brasília, Brasília, DF Brazil; 2 Instituto René Rachou, Fundação Oswaldo Cruz – FIOCRUZ-Minas, Belo Horizonte, MG Brazil; 3 Instituto de Tecnologia em Imunobiológicos Bio-Manguinhos – FIOCRUZ, Rio de Janeiro, RJ Brazil; 4 Programa Nacional de Imunizações – Secretaria de Vigilância em Saúde, Ministério da Saúde, Brasília, DF Brazil; 5 Grupo Integrado de Pesquisas em Biomarcadores, Instituto René Rachou, Fundação Oswaldo Cruz, FIOCRUZ-Minas, Avenida Augusto de Lima, 1715 Barro Preto, Belo Horizonte, 30190-002 Brazil

**Keywords:** Immunomodulatory therapy, Yellow fever vaccine, Neutralizing antibodies, Cellular immunity, Rheumatoid arthritis

## Abstract

**Background:**

The 17DD-yellow fever (YF) vaccine induces a long-lasting protective immunity, resulting from humoral and cellular immunological memory. The treatment of rheumatoid arthritis (RA) patients with disease-modifying anti-rheumatic drugs (DMARD) may affect pre-existing 17DD-vaccine protective immunity and increase the risk of acquiring YF infection. Our goal was to determine whether DMARD would affect the duration of YF-specific protective immunity in RA patients.

**Methods:**

A total of 122 RA patients, previously immunized with the 17DD-YF vaccine (1–5, 5–9, and ≥ 10 years) and currently under DMARD therapy, were enrolled in the present investigation. Immunomodulatory therapy encompasses the use of conventional synthetic DMARD alone (csDMARD) or combines with biological DMARD (cs+bDMARD). A total of 226 healthy subjects were recruited as a control group (CONT). Neutralizing antibody responses were measured by a plaque-reduction neutralization test (PRNT), and cellular immunity was evaluated by an in vitro 17DD-YF-specific peripheral blood lymphoproliferative assay.

**Results:**

The data demonstrated that csDMARD therapy did not affect the duration of protective immunity induced by the 17DD-YF vaccine compared to that of CONT, as both presented a significant time-dependent decline at 10 years after vaccination. Conversely, cs+bDMARD therapy induced a premature depletion in the main determinants of the vaccine protective response, with diminished PRNT seropositivity levels between 5 and 9 years and impaired effector memory in CD8^+^ T cells as early as 1–5 years after 17DD-YF vaccination.

**Conclusions:**

These findings could support changing the vaccination schedule of this population, with the possibility of a planned booster dose upon the suspension of bDMARD in cases where this is allowed, even before 10 years following 17DD-YF vaccination. The benefit of a planned booster dose should be evaluated in further studies.

**Trial registration:**

RBR-946bv5. Date of registration: March 05, 2018. Retrospectively registered

## Background

Rheumatoid arthritis (RA) is one of the most prevalent chronic autoimmune diseases, and it mainly affects the peripheral joints and promotes synovitis, which may lead to cartilage damage and bone erosion [1]. The prevalence of RA ranges from 0.40 to 1.60% and 0.46 to 1.00% in Latin America [2, 3] and Brazil [4, 5], respectively.

In recent decades, significant advances in RA clinical and therapeutic approaches have been reported worldwide, encompassing the use of conventional synthetic and biological strategies, such as pathway inhibitors/antagonists. While effective for controlling RA activity, these immunomodulatory therapies may affect pre-existing immunity to infectious diseases. In a scenario in which immunizations are effective to elicit protective immunity, it becomes relevant to understand the impact that disease-modifying anti-rheumatic drugs (DMARD) has on correlates of protection acquired upon vaccination [6, 7]. The ability of DMARD to modify or affect pre-existing vaccine-induced protective immunity, including the function of memory T and B cells and, as a consequence, yellow fever (YF)-specific neutralizing antibody levels, has already been reported [8]. There is a paucity of data available regarding the impact of DMARD on the duration of YF vaccine-induced immunological memory developed by RA patients.

The recent YF outbreaks in Angola (2016) and Brazil (2017/2018) [9, 10] brought about a relevant question regarding the impact that DMARD may have on RA patients previously immunized with the 17DD-YF vaccine. Assessing the duration of the immune responses triggered by YF vaccines can provide insights into elucidating the vulnerability to YF infection of RA patients under DMARD therapy.

In this context, the aim of the present study was to verify whether conventional synthetic or biological DMARD impact the cellular and humoral immunological memory of RA patients previously immunized with the 17DD-YF vaccine. These findings may be useful when making clinical decisions regarding YF vaccination in RA patients.

## Methods

### Subjects

Between September 17, 2014, and December 06, 2016, 136 adult patients (≥ 18 years) who met ACR classification criteria for RA were enrolled in this open-label, parallel cohort, single-center study. Patients had received a single dose of the 17DD-YF vaccine and time after vaccination estimated according to their vaccination card records; some patients have been inadvertently vaccinated before starting the DMARD therapy; all patients were residents of the metropolitan area of Brasilia, DF, Brazil, and received medical care at the University Hospital of Brasilia, University of Brasilia. Fifteen patients were excluded due to their 17DD-YF vaccination records showing < 1 year, > 30 years (*n* = 12), or missing data (*n* = 3). The final RA group comprised 121 subjects, 113 females and 9 males, aged 23 to 86 years, categorized into two subgroups based on whether they were under immunotherapy with conventional synthetic disease-modifying anti-rheumatic drugs (csDMARD, *n* = 73) or under combined immunotherapy with csDMARD plus biological disease-modifying anti-rheumatic drugs (cs+bDMARD, *n* = 48). The csDMARD and cs+bDMARD subgroups were further segregated according to the time after 17DD-YF vaccination, as follows: 1–5 years, > 5–9 years, and ≥ 10 years. Details regarding the demographic features, clinical records, and immunomodulatory therapy dosages are provided in Table 1.

**Table 1:** Demographic features, clinical records, and immunomodulatory therapy of the AR population

Parameters	csDMARD *n* = 73			cs+bDMARD *n* = 48		
	1–5 years *n* = 18	> 5–9 years *n* = 37	≥ 10 years *n* = 18	1–5 years *n* = 10	> 5–9 years *n* = 25	≥ 10 years *n* = 13
Gender (F/M)	17/01	35/02	15/03	09/01	23/02	13/00
Age (years)	58 (31–78)	53 (26–86)	49 (23–81)	55 (28–75)	60 (40–82)	54 (29–80)
Disease duration (months)	99 (12–432)	114 (12–348)	84 (12–480)	210 (96–298)	144 (72–370)	168 (96–288)
csDMARD dose						
MTX (2.5– 25 mg/week)	17 mg (14/18)	16 mg (35/37)	18 mg (12/18)	23 mg (05/10)	17 mg (14/25)	17 mg (04/13)
LEF (standard dose/day)	20 mg (06/18)	20 mg (19/37)	20 mg (09/18)	20 mg (05/10)	20 mg (12/25)	20 mg (06/13)
SSZ (1000–3000 mg/day)	1500 mg (02/18)	–	2300 mg (03/18)	–	1000 mg (02/25)	1200 mg (05/13)
AML (150–400 mg/day)	275 mg (04/18)	340 mg (05/37)	–	–	150 mg/day (01/25)	275 mg/day (02/13)
AZA (2–3 mg/kg/day)	–	–	–	150 mg (01/10)	–	–
CYC (3–5 mg/kg/day)	–	–	–	–	–	200 mg (01/13)
bDMARD dose						
ADA (standard dose/eow)	–	–	–	40 mg (01/10)	40 mg (02/25)	40 mg (01/13)
CTZ (standard dose/month)	–	–	–	400 mg (02/10)	–	400 mg (01/13)
ETN (standard dose/week)	–	–	–	50 mg (05/10)	50 mg (05/25)	50 mg (04/13)
GOL (standard dose/month)	–	–	–	–	50 mg (02/25)	50 mg (03/13)
IFX (3–5 mg/kg/e8w)	–	–	–	300 mg (01/10)	233 mg (06/25)	–
TCZ (8 mg/kg/month)	–	–	–	–	496 mg (05/25)	480 mg (02/13)
ABT (10 mg/kg/month)	–	–	–	–	750 mg (03/25)	750 mg (01/13)
RTX (standard dose/e6m)	–	–	–	1000 mg (01/10)	1000 mg (02/25)	1000 mg (01/13)
GC						
PDN (2.5–40 mg/day)	7.5 mg (04/18)	13.6 mg (06/37)	8.2 mg (07/18)	6.7 mg (03/10)	10.8 mg (06/25)	7.0 mg (05/13)

Age is expressed as median (min-max). Disease duration in months is expressed as median (min-max). Immunomodulatory therapeutic dosages are provided for each drug and as mean dose/group

*csDMARD* conventional synthetic disease-modifying anti-rheumatic drugs, *cs+bDMARD* combined conventional synthetic and biological disease-modifying anti-rheumatic drugs, *CG* glucocorticoid, *F* female, *M* male, *MTX* methotrexate, *LEF* leflunomide, *SSZ* sulfasalazine, *AML* anti-malarial drugs (hydroxychloroquine and chloroquine phosphate), *AZA* azathioprine, *CYC* ciclosporin, *ADA* adalimumab, *CTZ* certolizumab, *ETN* etanercept, *GOL* golimumab, *IFX* infliximab, *TCZ* tocilizumab, *ABT* abatacept, *RTX* rituximab, *PDN* prednisone, *eow* every other week, *e8w* every 8 weeks, *e6m* every 6 months

**Fig. 1:**
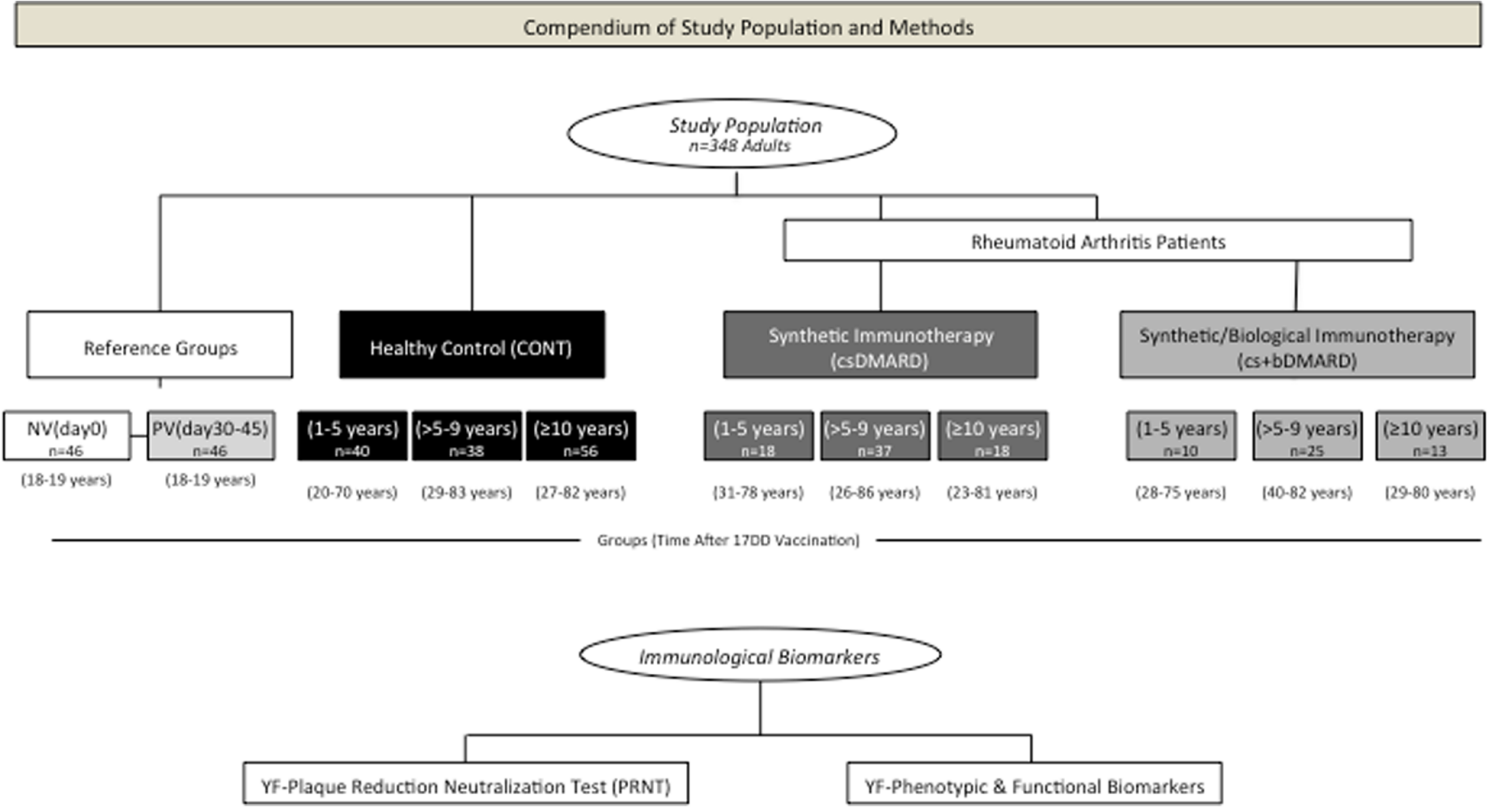
Compendium of the study population. A total of 348 adults were enrolled in the present investigation. One hundred and twenty-one adult RA patients with previous records of 17DD-YF vaccination were enrolled. Patients were first categorized into two subgroups, referred to as synthetic immunotherapy (csDMARD) or combined immunotherapy (cs+bDMARD) based on whether they were under current treatment with DMARDs or DMARDs combined with TNF-α inhibitors (adalimumab/ADA, certolizumab/CTZ, etanercept/ETN, golimumab/GOL, or infliximab/IFX), IL-6 antagonists (tocilizumab/TCZ), T lymphocyte co-stimulation modulators (abatacept/ABT), or anti-B-cell mAbs (rituximab/RTX); the patients were further categorized according to the time after 17DD vaccination as follows: csDMARD (1–5 years), csDMARD (> 5–9 years), csDMARD (≥ 10 years), and cs+bDMARD (1–5 years), cs+bDMARD (> 5–9 years), cs+bDMARD (≥ 10 years). The control group of the healthy subjects included 226 participants categorized into five subgroups referred as non-vaccinated subjects NV(day0), PV(day30–45), and three groups of volunteers, categorized according to the time after 17DD-YF vaccination and referred to as CONT(1–5 years), CONT(> 5–9 years), and CONT(≥ 10 years). Detailed descriptions of the study groups are provided in the “Methods” section. Immunological biomarker analyses, including YF plaque-reduction neutralization test (PRNT) and YF phenotypic and functional biomarkers, were performed for each participant

The control group of healthy subjects included 226 volunteers, 121 males and 59 females; the subjects were aged 18–82 years and categorized into five subgroups referred to as non-vaccinated subjects NV(day0) and primary vaccinated PV(day30–45) and three groups of healthy controls (CONT); the controls were categorized according to the time after their 17DD-YF vaccination: CONT(1–5 years), CONT(> 5–9 years), and CONT(≥ 10 years). Whole blood samples were collected from each volunteer: 5 mL without anticoagulant for the plaque-reduction neutralization test (PRNT) and 20 mL in heparin to isolate peripheral blood mononuclear cells (PBMC) for analyses of cellular immunity. A detailed compendium of the study population and methods are provided in Fig. 1.

This study was approved by the Ethics Committee for studies with human subjects at Instituto René Rachou FIOCRUZ (CPqRR # 180911). All subjects gave written informed consent in accordance with the Declaration of Helsinki.

### YF-neutralizing antibody test (PRNT)

The 17DD-YF-neutralizing antibody test (PRNT) was performed as previously described [11, 12]. The assays were carried out at Laboratório de Tecnologia Virológica, Bio-Manguinhos (LATEV, FIOCRUZ-RJ, Brazil), and the results are expressed as a reverse of the samples’ dilution. The samples were considered seropositive when the PRNT levels were higher than the serum dilution 1:50.

### Analysis of cellular immunity

PBMC (1.0 × 10^6^/well) were incubated for 144 h at 37 °C in a 5% CO_2_ humidified atmosphere, in the absence (Control/CC) or presence of 17DD-YF antigen (17DD-YF Ag), as described previously [13]. Following the long-term incubation, the PBMC were stained with live/dead dye and a cocktail of monoclonal antibodies (mAbs), including anti-CD4/(RPA-T4)/FITC, anti-CD8/(SK1)/PerCP-Cy5.5, anti-CD27/(M-T271)/PE, anti-CD45RO/(UCHL1)/PE-Cy, anti-CD3/(SK7)/APC-Cy7, anti-IgD/(IA6-2)/FITC, anti-CD27/(M-T271)/PE, and anti-CD19/(HIB19)/PerCP for the analysis of the T and B cell phenotypic memory status.

In parallel, PBMC were stained for the functional analysis of T and B cells. Cells were first incubated with anti-CD3/(UCHT1)/Qdot605, anti-CD4/(GK1.5)/APCe-Fluor780, anti-CD8/(SK1)/PerCP, and anti-CD19/(HIB19)/Alexa-Fluor700. Then, surface-stained PBMC were subjected to a fix/perm procedure and stained with anti-IFN-γ/(clone B27)/Alexa-Fluor488, anti-IL-5/(JES1-39D10)/PE, anti-IL-10/(JES3-19F1)/APC, and anti-TNF-α/(clone MAb11)/PE-Cy7. After staining, cells were fixed, and acquisition was carried out on an LSR Fortessa Flow Cytometer.

A total of 100,000 events were acquired per sample, and gating strategies were employed for phenotypic and functional memory using the FlowJo software, version 9.3.2, as previously described [13]. Four memory T cell subsets [naïve/(NCD4 and NCD8)/CD27^+^CD45RO^−^, early effector memory/(eEfCD4 and eEfCD8)/CD27^−^CD45RO^−^, central memory/(CMCD4 and CMCD8)/CD27^+^CD45RO^+^, and effector memory/(EMCD4 and EMCD8)/CD27^−^CD45RO^+^] and three memory B cell subsets [naïve/(NCD19)/CD27^−^IgD^+^, non-classical memory/(nCMCD19)/CD27^+^IgD^+^, and classical memory/(CMCD19)/CD27^+^IgD^−^] were quantified. Cytokine^+^ cells were also quantified (TNF-α, IFN-γ, IL-10, and IL-5 for T cells and TNF-α, IL-10, and IL-5 for B cells). The results were reported as the 17DD-YF Ag/CC index, computed as the frequency of cells observed in the 17DD-YF stimulated culture (17DD-YF Ag) divided by the respective control culture (CC). The characterization of phenotypic and functional features of PBMC has been performed after 17DD-YF-specific in vitro stimuli, and the results are expressed as stimulation index, taking the results from the control culture intrinsic for the same individual as a baseline.

### Multiparameter data mining strategies

Data analyses were carried out employing a set of strategies including conventional statistical, biomarker signature analysis, Venn diagram assembling, and overlaid signature curves.

Conventional statistical approaches were used for comparative analysis with the reference groups NV(day0) and PV(day30–45). For this purpose, the mean value of each study group (CONT, csDMARD, and cs+bDMARD) was compared with the 95% CI of the reference groups [NV(day0) and PV(day30–45)]. The differences demonstrated by the mean values outside the 95% CI were considered significant (*p* < 0.05) and highlighted by letters “a” and “b” compared to NV(day0) or PV(day30–45). Biomarker signature analysis was carried out as described previously [14], using the global median value of 17DD-YF Ag/CC index for each biomarker as the cut-off to define “low” or “high” 17DD-YF Ag/CC index. The biomarker signatures of NV(day0) and PV(day30–45) were overlapped as the reference curves, and those biomarkers for which more than 50% of samples were above the cut-off index were selected for further identification of biomarkers upregulated selectively by the 17DD-YF vaccine using the Venn diagram analysis (http://bioinformatics.psb.ugent.be/webtools/Venn/). The selected set of biomarkers identified early after 17DD-YF vaccination in the PV(day30–45) group was underscored in a bold font format. These attributes were employed for comparative analyses among those biomarkers by overlaid signature curves in which the 50th percentile defined significant differences for each study group (CONT, csDMARD, and cs+bDMARD) at distinct time points after 17DD-YF vaccination.

## Results

### Early decrease of 17DD-YF-neutralizing antibodies is observed in RA patients undergoing combined synthetic/biological immunomodulatory therapy

**Fig. 2:**
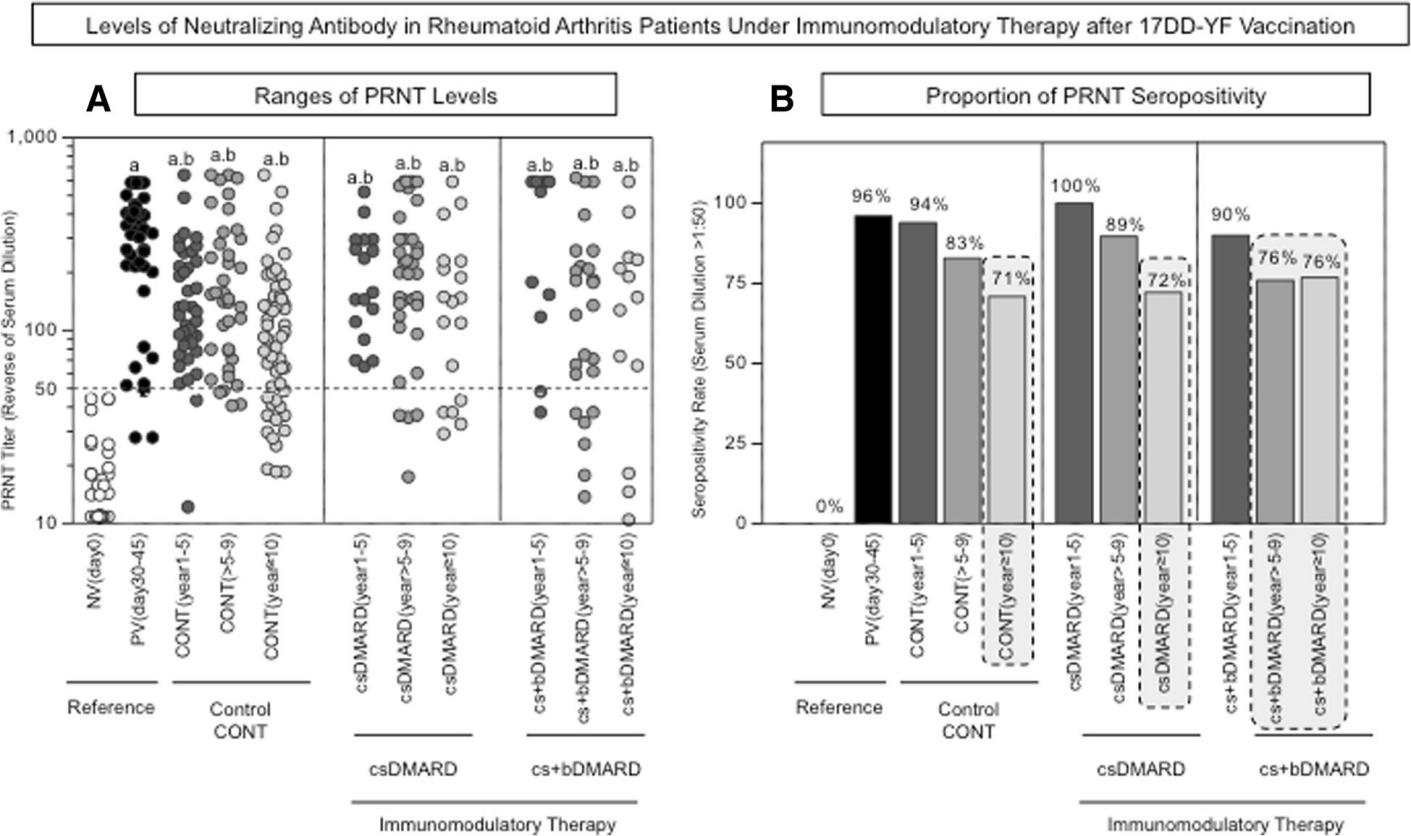
Levels of neutralizing antibodies in patients undergoing immunomodulatory therapy after 17DD-YF vaccination. PRNT was performed as described previously by Simões et al. [11]. The data are reported as **a** ranges of PRNT levels and **b** the proportion of PRNT seropositivity (serum dilution > 1:50) for the reference groups [NV(day0) and PV(day30–45)], and nine study groups are referred to as CONT(1–5 years), CONT(> 5–9 years), CONT(≥10 years), csDMARD(1–5 years), csDMARD(> 5–9 years), csDMARD(≥10 years), cs+bDMARD(1–5 years), cs+bDMARD(> 5–9 years), and cs+bDMARD(≥ 10 years). Significant differences in PRNT levels were highlighted by letters “a” and “b” compared to NV(day0) or PV(day30–45), respectively. Seropositivity rates below 80% were considered critical and were underscored by gray background rectangles

The analysis of PRNT levels is presented in Fig. 2. Data are reported as ranges of PRNT levels and proportion of PRNT seropositivity (serum dilution > 1:50). The results demonstrated a decrease of PRNT levels over time after 17DD-YF vaccination in all study groups (CONT, csDMARD, and cs+bDMARD) compared to the reference group PV(day30–45) (Fig. 2a). The PRNT seropositivity rate reaches critical values in CONT (71%) and csDMARD (72%) at 10 years after vaccination (Fig. 2b). Conversely, data demonstrated that combined immunotherapy has a deleterious impact on the PRNT seropositivity rate. In fact, in the cs+bDMARD group, the decrease in the PRNT seropositivity rate occurs earlier compared to CONT and sDMARD, reaching critical values (76%) at > 5–9 years after 17DD-YF vaccination (Fig. 2b).

### Distinct duration of 17DD-YF-specific phenotypic memory biomarkers is observed in RA patients upon immunomodulatory therapy

**Fig. 3:**
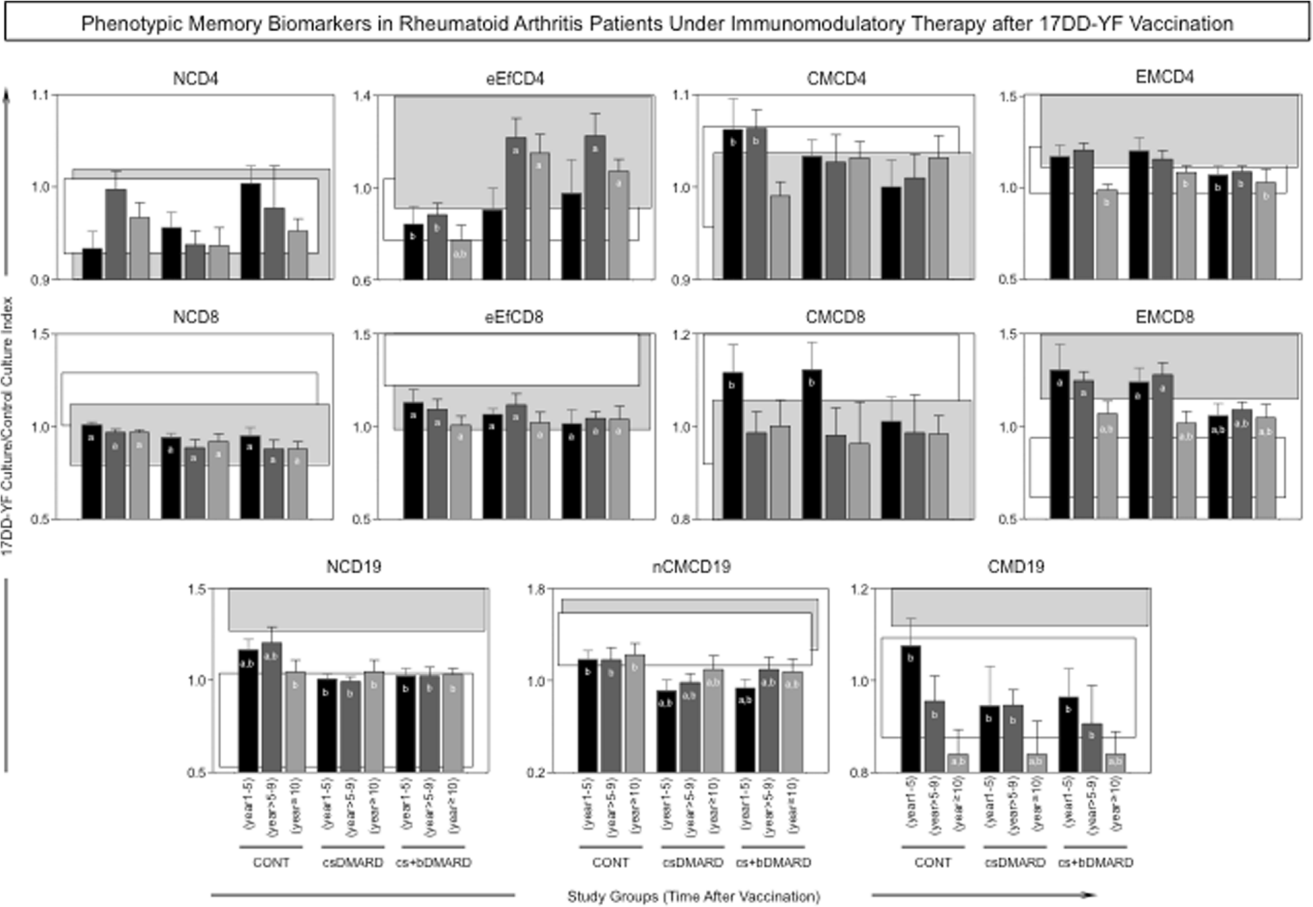
Phenotypic memory biomarkers in patients undergoing immunomodulatory therapy after 17DD-YF vaccination. An in vitro 17DD-YF-specific peripheral blood lymphoproliferative assay was employed for antigen recall. Flow cytometric assay were carried out for distinct memory T cell subsets, including naïve/(NCD4 and NCD8)/CD27^+^CD45RO^−^, early effector memory/(eEfCD4 and eEfCD8)/CD27^−^CD45RO^−^, central memory/(CMCD4 and CMCD8)/CD27^+^CD45RO^+^, and effector memory/(EMCD4 and EMCD8)/CD27^−^CD45RO^+^, along with memory B cell subsets, including naïve/(NCD19)/CD27^−^IgD^+^, non-classical memory/(nCMCD19)/CD27^+^IgD^+^, and classical memory/(CMCD19)/CD27^+^IgD^−^. The results are expressed as 17DD-YF Ag/CC index as described in the “Methods” section. Comparative analyses with the reference groups NV(day0) and PV(day30–45) were carried out using the mean value observed for each study group CONT(1–5 years), CONT(> 5–9 years), CONT(≥10 years), csDMARD(1–5 years), csDMARD(> 5–9 years), csDMARD(≥10 years), cs+bDMARD(1–5 years), cs+bDMARD(> 5–9 years), and cs+bDMARD(≥10 years) in comparison to the 95% CI of the reference groups, including NV(day0) (white rectangles) and PV(day30–45) (gray rectangles). Significant differences were highlighted by letters “a” and “b” compared to NV(day0) or PV(day30–45), respectively

The profile of 17DD-specific phenotypic memory biomarkers is shown in Fig. 3. Our data show that patients with RA undergoing immunomodulatory therapy (csDMARD or cs+bDMARD) presented a distinct overall profile of phenotypic memory biomarkers, characterized by increased levels of eEfCD4 and decreased levels of CMCD4, NCD19, and nCMCD19 compared to CONT. The cs+bDMARD group presented a particular decrease of EMCD4, CMCD8, and EMCD8 as early as 1–5 years after 17DD-YF vaccination compared to CONT and csDMARD (Fig. 3).

### Early decrease of 17DD-YF-specific functional memory biomarkers is observed in RA patients receiving synthetic/biological combined immunomodulatory therapy

**Fig. 4:**
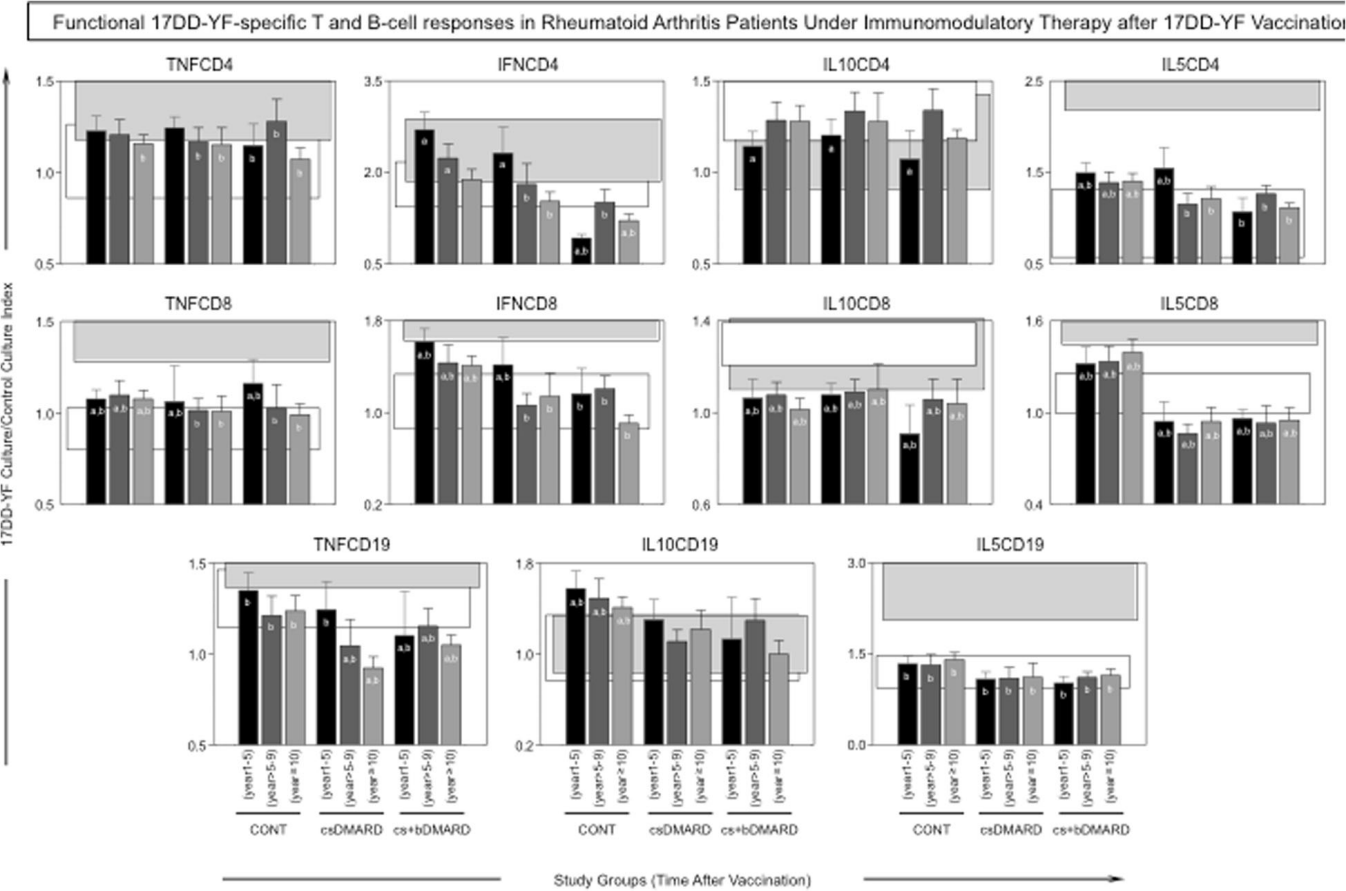
Functional biomarkers in patients undergoing immunomodulatory therapy after 17DD-YF vaccination. An in vitro 17DD-YF-specific lymphocyte proliferation assay was employed for antigen recall. Flow cytometric assay was carried out to identify functional T cell subsets, including cells producing TNF-α, IFN-γ, IL-10, and IL-5, as well as functional B cell subsets, including cells producing TNF-α, IL-10, and IL-5. The results are expressed as 17DD-YF Ag/CC index as described in the “Methods” section. Comparative analyses with the reference groups NV(day0) and PV(day30–45) were carried out using the mean value observed for each study group CONT(1–5 years), CONT(> 5–9 years), CONT(≥10 years), csDMARD(1–5 years), csDMARD(> 5–9 years), csDMARD(≥10 years), cs+bDMARD(1–5 years), cs+bDMARD(> 5–9 years), and cs+bDMARD(≥10 years) in comparison to the 95% CI of the reference groups, including NV(day0) (white rectangles) and PV(day30–45) (black rectangles). Significant differences were highlighted by letters “a” and “b” compared to NV(day0) or PV(day30–45), respectively

The profile of 17DD-specific phenotypic biomarkers is shown in Fig. 4. Patients with RA undergoing immunomodulatory therapy (csDMARD or cs+bDMARD) presented a distinct pattern of functional biomarkers, particularly exemplified by decreased levels of IL-5CD8 and IL-10CD19 compared to CONT. Noteworthy was the decrease of IFNCD4, IFNCD8, TNFCD4, and TNFCD19, along with lower levels of IL-5CD4 observed in the cs+bDMARD group as early as 1–5 years after 17DD-YF vaccination compared to CONT and csDMARD (Fig. 4).

### 17DD-YF-specific memory biomarker signatures in RA patients undergoing immunomodulatory therapy

**Fig. 5:**
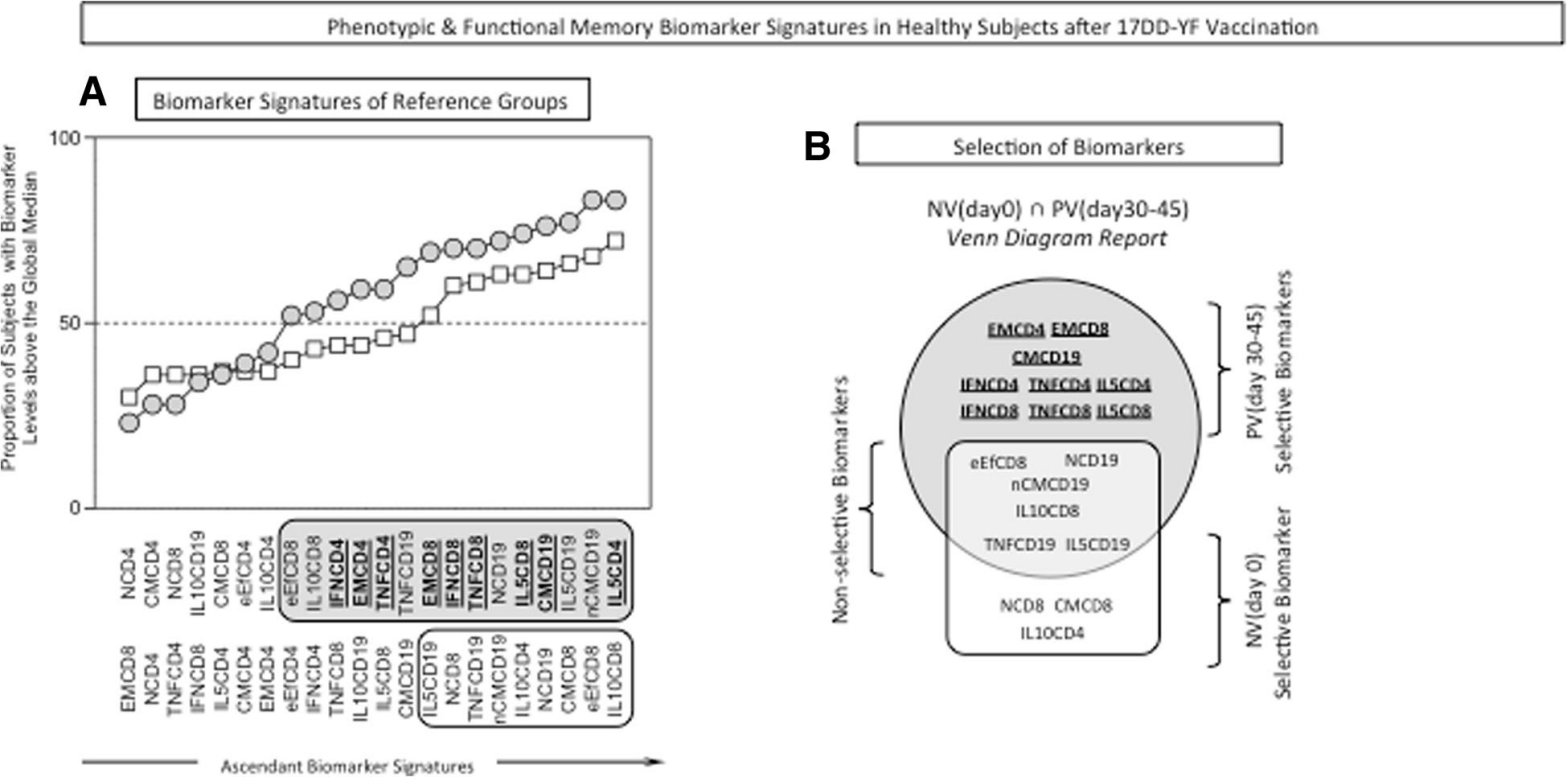
Biomarker signatures of healthy subjects before and after 17DD-YF vaccination. Biomarker signature analysis was carried out as described previously by Luiza-Silva et al. [14] as described in the “Methods” section. **a** The biomarker signatures of NV(day0) [white squares] and PV(day30–45) [gray circles] were overlapped as the reference curves, and those biomarkers for which more than 50% of samples were above the cut-off index were selected for further identification of biomarkers upregulated selectively by the 17DD-YF vaccine. **b** Venn diagram analysis was employed to identify the set of biomarkers selectively increased in the PV(day30–45) group, representing those biomarkers elicited early after 17DD-YF vaccination. These attributes were underscored in a bold font format and selected for further comparative analysis of duration of 17DD-YF-specific responses after vaccination among the study groups

The biomarker signature has been proposed previously [14] as a reliable approach to characterize the overall profile of immune responses in patients vaccinated with 17DD vaccines. This approach allows the identification of the most relevant biomarkers among a range of attributes. For this purpose, we performed a comparative analysis between the biomarker signatures of the reference groups NV(day0) and PV(day30–45) to identify biomarkers selectively elicited early after vaccination (Fig. 5a). Using this multiparameter approach and Venn diagrams (Fig. 5b), we identified a set of 17DD-YF-specific biomarkers useful for further monitoring the memory signature of the study groups (CONT, csDMARD, and cs+bDMARD) over time after vaccination. A set of nine phenotypic and functional biomarkers (EMCD4, EMCD8, CMCD19, IFNCD4, TNFCD4, IL-5CD4, IFNCD8, TNFCD8, and IL-5CD8) was identified as selectively predominant in the biomarker signatures of the PV(day30–45) (Fig. 5b).

**Fig. 6:**
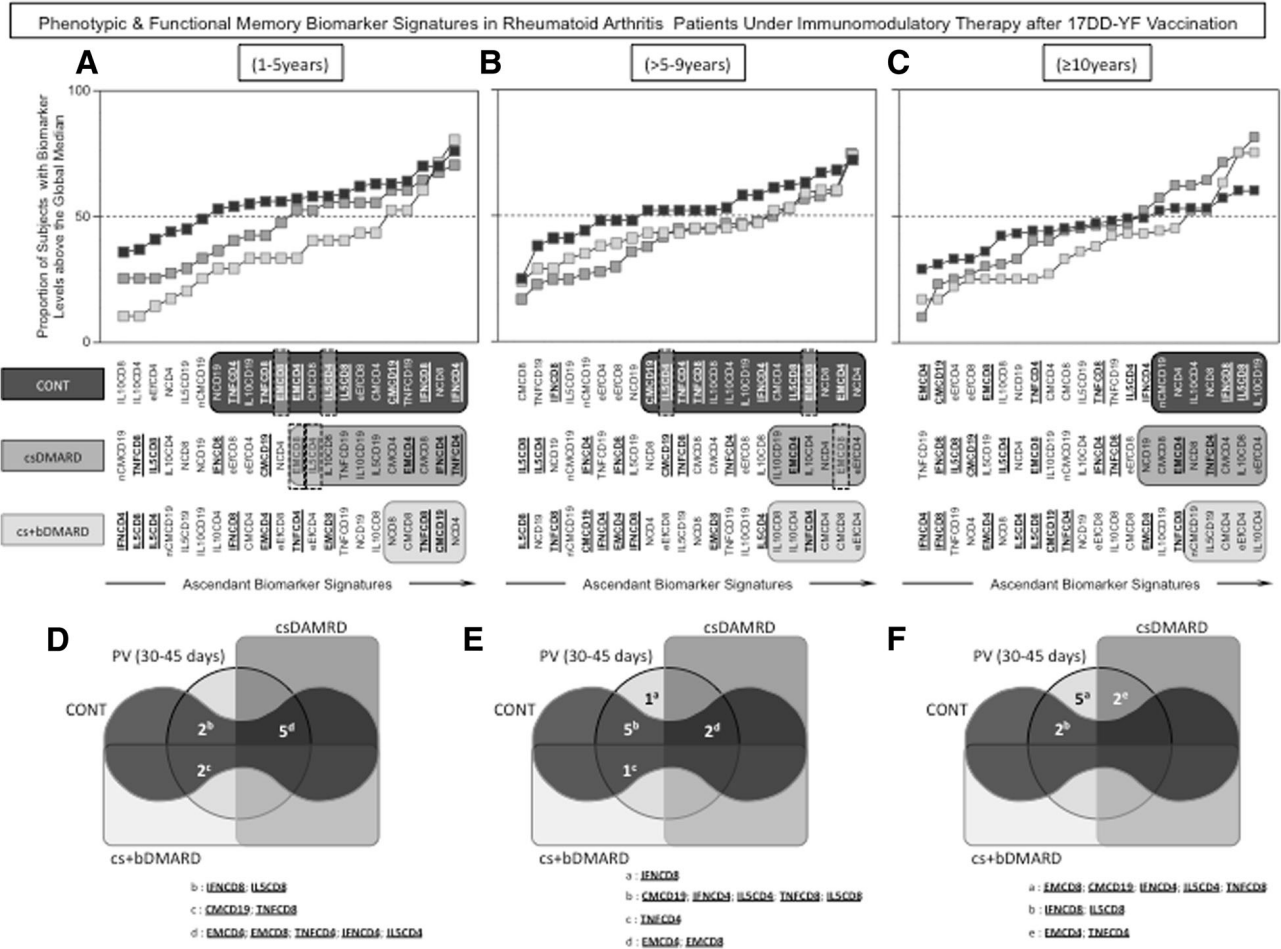
Phenotypic and functional memory biomarker signatures in patients undergoing immunomodulatory therapy after 17DD-YF vaccination. Overlaid biomarker signatures were employed for comparative analysis among groups at distinct time points after vaccination, including **a** 1–5 years, **b** > 5–9 years, and **c** ≥ 10 years. Those biomarkers above the 50th percentile observed in each time point were highlighted by black/gray background rectangles, to underscore those observed in CONT (black background), csDMARD (dark gray background), and cs+bDMARD (light gray background). The set of nine phenotypic and functional biomarkers (EMCD4, EMCD8, CMCD19, IFNCD4, TNFCD4, IL-5CD4, IFNCD8, TNFCD8, and IL-5CD8) selected for comparative analysis of duration of 17DD-YF memory after vaccination is tagged by a bold font format. Special attention is given to EMCD8 and IL-5CD4 (dashed frame), which were previously reported as the top two biomarkers for monitoring immunological memory to the 17DD-YF vaccine [15]. **b** Venn diagrams were constructed to further demonstrate the shared biomarkers observed among the reference [PV(day30–45)] and study groups. Letter “**a**” represents the biomarkers selectively observed in the reference group [PV(day30–45)]. Letters “**b**,” “**c**,” and “**d**” represent those biomarkers commonly observed in PV(day30–45)∩CONT, PV(day30–45)∩CONT∩cs+bDMARD, and PV(day30–45)∩CONT∩csDMARD, respectively, and letter “**e**” represents the biomarkers shared between PV(day30–45)∩csDMARD. Letter “**d**” represents the biomarkers shared between PV(day30-45)∩CONT; PV(day30-45)∩CONT∩csDMARD and PV(day30–45)∩CONT∩cs+bDMARD in the subgroup 1-5 years; Letter “**e**” represents the biomarkers shared between PV(day30-45)∩CONT; PV(day30-45)∩CONT∩csDMARD; PV (day30–45)∩CONT∩cs+bDMARD and those biomarkers seen exclusively in PV(day30-45) in the subgroup > 5-9 years. Letter “**f**” represents the biomarkers shared between PV(day30-45)∩CONT; PV(day30-45)∩csDMARD; and those biomarkers seen exclusively in PV(day30-45) in the subgroup ≥10 years

This set of biomarkers was then employed to follow up with the phenotypic/functional memory signatures among the biomarkers frequently observed above the 50th percentile over time after 17DD-YF vaccination for each study group (CONT, csDMARD, and cs+bDMARD) (Fig. 6). Among these biomarkers, special attention was given to EMCD8 and IL-5CD4, previously reported as the top two biomarkers to monitor immunological memory to the 17DD-YF vaccine [15]. Overlaid biomarker signatures were plotted for comparative analysis among groups at distinct time points after vaccination, including 1–5 years—Fig. 6a, > 5–9 years—Fig. 6b, and ≥10 years—Fig. 6c. The results demonstrated that CONT and csDMARD presented a progressive decrease in the number of biomarkers above the 50th percentile, reaching a critical profile with an absence of EMCD8 at 10 years after vaccination. Conversely, the cs+bDMARD group displayed an overall shortage on the number of biomarkers above the 50th percentile, with an absence of EMCD8 as early as 1–5 years after 17DD-YF vaccination.

Venn diagrams were constructed and corroborated the finding that RA patients undergoing combined immunomodulatory therapy did not share either of the top two biomarkers (EMCD8 and IL-5CD4), considered correlates of protection elicited by the 17DD vaccination, with the PV(day30–45) reference group. The CONT and csDMARD groups presented sustained relevant levels of EMCD8 up to > 5–9 years but showed a critical decrease in this cell subset at 10 years after 17DD-YF vaccination. On the other hand, the cs+bDMARD group displayed a premature loss of the top two biomarkers as early as 1–5 years after vaccination.

## Discussion

This is an innovative investigation that has been performed to evaluate the impact of immunomodulatory therapy on the duration of the YF vaccine response in RA patients who were previously given the 17DD vaccine.

In general, both immunomodulatory therapies (csDMARD or cs+bDMARD) induced an increase in eEfCD4; a decrease in CMCD4, NCD19, and nCMCD19; and diminished IL-5CD8 and IL-10CD19 compared to CONT. These cell subsets have not been nominated as the most relevant correlates of protection in adults that received the 17DD-YF vaccine [15]. Overall, less significant changes in cellular immunity were observed in RA patients undergoing therapy with csDMARD, who presented an overall similar profile to CONT. Conversely, the evidence demonstrated that therapy with cs+bDMARD has a strong impact on vaccine-induced 17DD-YF-specific memory T and B cell responses. Notably, RA patients receiving cs+bDMARD presented a decrease in PRNT levels at > 5–9 years post-vaccination and a decrease in cellular memory-related markers (EMCD4, CMCD8, EMCD8, IFNCD4, IFNCD8, TNFCD4 and TNFCD19, and IL-5CD4) as early as 1–5 years after vaccination compared to the CONT and csDMARD groups. In association with the PRNT, EMCD8 and IL-5CD4 were recently nominated as the most relevant biomarkers to follow up with the immune response over time after the 17DD-YF vaccination [15]. The present results corroborate these two correlates of memory response upon 17DD-YF vaccination.

Neutralizing antibodies have long been known to provide protection against challenge with a wild-type virus [16]. The protective role of neutralizing antibodies induced by the YF vaccine has been estimated from dose-response studies carried out in experimental models that were challenged with a virulent YF virus after immunization [17, 18]. In this sense, the PRNT is considered the most sensitive and specific assay for the quantification of neutralizing antibodies, as well as the reference method for assessing the protective immune response after vaccination [11]. Neutralizing antibodies are induced within 30 days in approximately 98% healthy adults that received the 17D and 17DD-YF vaccines [19]. Although the neutralizing antibodies are long-lasting, a progressive decrease in the PRNT levels is observed over time after YF vaccination, with approximately 25–30% of vaccine recipients presenting seroreversion after 10 years of vaccination, suggesting the need of a booster dose to maintain the protective immunity [13, 20, 21]. Our findings corroborate these studies, as demonstrated by the evidence that both CONT and csDMARD groups presented a decrease in PRNT levels and in seropositivity rates over time, reaching critical values after 10 years post-vaccination. The earlier loss of humoral response triggered by cs+bDMARD was confirmed by the critical decrease in PRNT seropositivity rate to 76%, observed at > 5–9 years post-vaccination in the RA patients undergoing conventional synthetic plus biological immunotherapy schemes; this outcome contrasts with the standard decline observed in CONT and csDMARDs after 10 years of 17DD-YF vaccination. In the light of this information, the possibility of a planned booster dose upon suspension of bDMARD should be considered as a strategy to overcome the impaired levels of YF-specific memory-related responses in specific RA cases.

Several studies have reported in detail the development of the cellular immune response after YF vaccination and characterized the phenotypic and functional changes that contribute to the establishment of effector memory [13, 14, 22–25]. Both CD4^+^ and CD8^+^ T cells respond strongly to the YF vaccine [16]. It is expected that CD4^+^ T cells would act primarily to support the production of neutralizing antibody responses. CD8^+^ T cells also respond upon YF vaccination and are considered necessary to recognize and eliminate virus-infected cells. Following the initial peak of CD8^+^ T cells that occurs early after YF vaccination, the CD8^+^ T cells begin to differentiate into long-lived memory with a polyfunctional phenotype. Effector memory CD8^+^ T cells have been considered one of the top two biomarkers for monitoring the long-lasting cellular immunity triggered by the 17DD-YF vaccine [15]. In the present study, the cs+bDMARD group displayed an early loss of cellular memory to 17DD-YF vaccine, as demonstrated by reduced levels of CMCD4, CMCD8, EMCD4, and EMCD8.

When analyzing the cytokine profile, it is observed that the cs+bDMARD group presents an early and marked decrease in IFNCD4, IFNCD8, TNFCD4, TNFCD19, and IL5CD4, important markers of the immune response triggered by the YF-17DD vaccine. Increased levels of IFN-γ, TNF-α, and IL-5 have been reported previously [14, 26, 27]. Particularly, TNF has been suggested as a biomarker with a pivotal role in the 17DD-YF vaccine-induced immunity. The prominent participation of TNF produced by neutrophils, monocytes, and CD4^+^ T cells is necessary for the establishment of protective immunity following YF-17DD primary vaccination, free of adverse events [27]. In this sense, it has been demonstrated that children not responding to 17DD-YF primary vaccination presented a deficiency in the synthesis of TNF by neutrophils and monocytes [14]. Moreover, it has been shown that a decreased production of TNF, mainly by monocytes and CD4^+^ T cells, is associated with the occurrence of severe adverse events after 17DD-YF primary vaccination [28].

In a systematic review evaluating the efficacy of vaccines in patients using immunosuppressant therapy, it was noted that the use of TNF-α antagonists in combination with methotrexate was associated with a reduction of immunogenicity of influenza and pneumococcal vaccines [8, 29]. Similarly, the use of RTX also led to a similar deleterious impact on the vaccine response to influenza and pneumococcal vaccines [29–32]. On the other hand, in general, AML, SSZ, and LEF did not alter the immunogenicity of these vaccines [31, 32]. These findings are consistent with those found in this study.

The strengths of the study are the simultaneous analysis of cellular and humoral analysis of RA patients previously immunized with the 17DD-YF vaccine and currently using immunosuppressive drugs. Moreover, the csDMARDs and cs+bDMARDS groups have a considerable sample size, differing from previous investigations. The present study has some limitations, considering that it is a cross-sectional investigation that enrolled a convenient sample of RA patients, several of them with previous therapeutic schemes, making it difficult to analyze the impact of each class of DMARD. Furthermore, as this is a cross-sectional study, it does not allow the analysis of the temporal dynamics of the immune response induced by the 17DD-YF vaccine in each individual.

Together, our findings showed that RA patients undergoing treatment with cs+bDMARD have a shorter duration of 17DD-YF vaccine-induced immunity. Based on these results, we suggest that a planned booster dose should be provided to RA patients, especially those residents or travelers to YF-endemic regions. The Brazilian Societies of Rheumatology, Tropical Medicine and Immunization have issued a technical note recommending YF vaccination for patients with immune-mediated rheumatic diseases who are at risk of YF, including those undergoing low immunosuppression or even those under cs+bDMARD therapy for whom the discontinuation of medication is allowed. [33] This vaccination strategy would boost YF-17DD immune responses and ensure safe vaccination, allowing the subsequent return of immunomodulatory therapy after safe immunization.

## Conclusions

csDMARD therapy did not affect the duration of protective immunity induced by the 17DD-YF vaccine compared to that of CONT, as both presented a significant time-dependent decline at 10 years after vaccination. Conversely, cs+bDMARD therapy induced a premature depletion in the main determinants of the vaccine protective response, with diminished PRNT seropositivity levels between 5 and 9 years and impaired effector memory in CD8+ T cells as early as 1–5 years after 17DD-YF vaccination.

These findings could support changing the vaccination schedule of this population, with the possibility of a planned booster dose upon the suspension of bDMARD in cases where this is allowed, even 10 years following 17DD-YF vaccination. The benefit of a planned booster dose should be evaluated in further studies.

## Abbreviations

e6m: Every 6 months
e8w: Every 8 weeks
eow: Every other week
ABT: Abatacept
ACR: American College of Rheumatology
ADA: Adalimumab
AML: Anti-malarial
AZA: Azathioprine
CC: Control culture
CM: Central memory
CONT: Control group
cs+bDMARD: Conventional synthetic plus biological DMARD
csDMARD: Conventional synthetic DMARD
CTZ: Certolizumab
CYC: Ciclosporin
DMARD: Disease-modifying anti-rheumatic drugs
eEf: Early effector memory
EM: Effector memory
ETN: Etanercept
GOL: Golimumab
IFX: Infliximab
LEF: Leflunomide
mAbs: Monoclonal antibodies
MTX: Methotrexate
N: Naïve
nCM: Non-classical memory
NV: Non-vaccinated
PBMC: Peripheral blood mononuclear cells
PDN: Prednisone
PRNT: Plaque-reduction neutralization test
PV: Primary vaccinated
RA: Rheumatoid arthritis
RTX: Rituximab
SSZ: Sulfasalazine
TCZ: Tocilizumab
TNF: Tumor necrosis factor
YF: Yellow fever
